# Evaluation of vascular cognitive impairment and identification of imaging markers using machine learning: a multimodal MRI study

**DOI:** 10.3389/fneur.2025.1505739

**Published:** 2025-05-29

**Authors:** Haoying He, Dongwei Lu, Sisi Peng, Jiu Jiang, Fan Fan, Dong Sun, Tianqi Sun, Zhipeng Xu, Ping Zhang, Xiaoxiang Peng, Ming Lei, Junjian Zhang

**Affiliations:** ^1^Department of Neurology, Zhongnan Hospital of Wuhan University, Wuhan, China; ^2^Department of Neuropsychology, Zhongnan Hospital of Wuhan University, Wuhan, China; ^3^Electronic Information School, Wuhan University, Wuhan, China; ^4^Department of Neurology, Third People's Hospital of Hubei Province, Wuhan, China; ^5^Department of Neurology, General Hospital of the Yangtze River Shipping, Wuhan, China

**Keywords:** vascular cognitive impairment, machine learning, magnetic resonance imaging, diffusion tensor imaging, imaging marker

## Abstract

**Background:**

Vascular cognitive impairment (VCI) is prevalent but underdiagnosed due to its heterogeneous nature and the lack of reliable diagnostic tools. Machine learning (ML) enhances disease evaluation by enabling accurate prediction and early detection from complex data. This study aimed to develop ML models to detect VCI using clinical data and multimodal MRI, and to explore the associations between imaging markers and cognitive function.

**Methods:**

The study enrolled 313 participants from Wuhan and surrounding areas, including 157 patients with VCI (age 62.38 ± 6.62 years, education 10.83 ± 3.00 years) and 156 cognitively normal individuals with vascular risk factors (age 59.93 ± 6.74 years, education 13.97 ± 3.19 years). An independent dataset of 82 participants was used for external validation. Clinical data, neuropsychological assessments, and MRIs (T1, T2-FLAIR, and DTI) were collected. After imaging processing and preliminary model selection, optimal models using various data modalities were constructed. Model reduction was undertaken to simplify models without sacrificing performance. SHapley Additive exPlanations and moDel Agnostic Language for Exploration and eXplanation were used for model interpretation.

**Results:**

The comprehensive final model integrating clinical and multimodal MRI measures achieved the best performance with eight input variables (AUC of 0.956, 95%CI 0.919–0.988 for internal and 0.919, 95%CI 0.866–0.966 for external validation). During external validation, DTI demonstrated more stable performance than T1 and T2-FLAIR imaging, highlighting its potential importance over conventional imaging markers. Key imaging markers, especially along the lateral cholinergic pathway, were highlighted for their importance in diagnosing VCI and understanding its manifestation.

**Conclusion:**

Our study developed and validated accurate ML models for VCI detection, emphasizing the importance of DTI. The identified imaging markers, particularly those derived from DTI, underscoring the potential in enhancing diagnostic accuracy and understanding cognitive impairments related to vascular changes.

## 1 Introduction

Vascular cognitive impairment (VCI) refers to a broad spectrum of cognitive impairment ranging from mild cognitive impairment to dementia, attributed to cerebrovascular pathologies and the burden of vascular risk factors (1). It is considered the second most prevalent form of cognitive impairment, potentially the most common in East Asia (2–4). Including cases of dementia with mixed pathology and white matter hyperintensities (WMHs), VCI accounts for 50–80% of all dementia cases (3). The impact of potential underlying neurodegenerative pathologies and various vascular pathologies on cognition varies greatly among individuals, complicating the encapsulation of VCI within a single diagnostic framework (5).

The criteria for diagnosing VCI include the presence of cognitive impairment, cerebral vascular injuries, and a predominant causal relationship between vascular burden and cognitive decline (2). For cognitive evaluation, “60-min” or “30-min” neuropsychological assessment protocols are recommended (1). However, these assessments are time-consuming, challenging in the presence of visual or hearing impairments, or dysgraphia, and subject to inter-rater and intra-rater variability (6). Only neurologists with substantial training and experience can conduct these assessments accurately. Besides, quantifying cerebral vascular burden and establishing causality between vascular burden and cognitive decline is difficult. The large interindividual variability in cerebral vascular burden and its relationship with cognitive status challenges the determination of causality. Simplistically categorizing individuals with high vascular burden as having VCI is inadequate. Moreover, the so called covert vascular brain injuries are prevalent in both cognitively impaired individuals and cognitively intact older adults, complicating the definition of vascular burden. Consequently, despite its prevalence, VCI is significantly underdiagnosed or diagnosed late in clinical practice, lacking reliable and straightforward diagnostic tools, especially for individuals with risk factors (7–9).

Brain MRI is deemed the “gold standard” for diagnosing VCI. Vascular lesions are primarily assessed visually; however, visual rating scales offer limited information, being insufficient both to accurately describe lesion distribution and to capture the heterogeneity inherent in VCI. Moreover, distinguishing patients with VCI from cognitive normal individuals using acknowledged imaging markers is challenging, as there are no defined thresholds of vascular burdens to indicate VCI. For instance, small vessel disease (SVD) is a primary cause of VCI, yet it remains unclear how the severity of imaging manifestations of SVD indicates the severity of cognitive impairment. Advanced imaging techniques, such as Diffusion Tensor Imaging (DTI), which evaluates microstructural integrity beyond visible injuries, is demonstrated to offer promising insights into cognitive profiles (3). DTI measures may serve as a more decisive predictor for cognitive deficits than other conventional markers (10). Further translation and quantification of images, alongside the integration of new imaging technologies, show promise in offering enhanced value for the interpretation of individual cognitive profiles (10).

Besides MRI findings, comprehensive VCI diagnostic criteria must also consider clinical vascular events and clinical information like demographics and medical histories (2, 11). In some guidelines, even a history of stroke or vascular risk factors burden may suffice when MRI is unavailable. Additionally, brain reserve has emerged as new evidence suggesting independent relationships between brain reserve, cerebral vascular injury, and cognition, not yet incorporated into current diagnostic criteria (12). Education and lifestyle factors, such as moderate exercise and smoking habits, are also critical for understanding an individual’s cognitive profile, especially considering recent lifestyle and environmental changes (13, 14).

Recent advances in machine learning (ML) technology have enabled the analysis of complex relationships in high-dimensional, multimodal data, increasingly used in clinical applications (15). While many ML models have been developed for Alzheimer’s disease, few have been explored for VCI (16, 17). Liu et al. developed radiomics-based diagnostic models for subcortical ischemic vascular cognitive impairment using a dataset of 116 participants (18). Wang et al. constructed VCI models based on white matter diffusion and cortical perfusion features in a sample of 113 participants, achieving an accuracy of 72.57% (19). Given VCI’s heterogeneous nature, there is a greater need for reliable and accurate ML models in VCI. Model Furthermore, the interplay between risk factors, quantified imaging findings, and cognition in VCI requires further investigation. In recent years, model interpretability has become a critical component of machine learning applications (20). Interpretation methods such as SHAP (SHapley Additive exPlanations) facilitate the identification of key predictors and provide insights into underlying mechanisms, enabling a better understanding of how specific variables contribute to model predictions (21). These approaches are particularly valuable in VCI, where the complex interactions and relative contributions of various risk factors for cognitive decline remain difficult to accurately define (22).

To address these needs, we developed ML models based on multimodal data, including conventional SVD imaging markers, detailed brain volume and cortical surface metrics from T1 images, DTI measures, and clinical information reflecting individuals’ vascular risk burden, clinical history, and brain reserve. We developed these models using data from a VCI cohort and subsequently validated them with an external dataset. We aimed to develop reliable models for distinguishing patients with VCI, elucidate the model’s interpretability, identify key contributors, and explore the associations between critical imaging markers in cortical and white matter (WM) regions and cognitive functions. Pharmacological treatment of VCI has primarily focused on cholinesterase inhibitors (22, 23). The key imaging markers identified in our study, especially those located along the lateral cholinergic pathway, may provide supporting evidence for the hypothesis of cholinergic pathway dysfunction in VCI and offer a potential neuroimaging basis for targeted therapeutic strategies (24). To this end, our models achieved high accuracy in VCI assessment by integrating these diverse measures and components, offering new insights into influential imaging markers and risk factors for better understanding cognitive profiles.

## 2 Materials and methods

### 2.1 Participants

In this study, multi-modal data was collected from 313 participants in the Zhongnan VCI cohort. These participants were consecutively recruited from the Department of neurology at Zhongnan hospital and multiple communities in Wuhan from 2020 to 2023, including 157 with VCI and 156 cognitive normal participants with risk factors.

The inclusion criteria were: (1) age between 45 and 75; (2) at least primary school education (6 years); (3) able to cooperate in completing neuropsychological assessments and MRI examinations; (4) patients with VCI should meet the Vascular Impairment of Cognition Classification Consensus Study (VICCCS) diagnostic criteria (1), including a cognitive impairment objectified with the neuropsychological assessments and presence of cerebrovascular (including ischemic and hemorrhagic injury, large and small vessel diseases, and hypoperfusion) diseases or vascular risk factors; (5) cognitive normal subjects should report no complain of cognitive impairment and present no cognitive impairment in neuropsychological assessments, and be burdened with risk factors, which were susceptible to cognitive impairment; (6) signing the informed consent form.

The exclusion criteria were: (1) a history of recent stroke (within 3 months); (2) other central neurological diseases; (3) other systemic diseases that may contribute to cognitive impairment; (4) visual impairment, hearing disorder or other impairments affecting the neuropsychological assessments; (5) MRI scans with serious artifacts from substantial movements.

The risk factors included hypertension, diabetes, hyperlipidemia, abdominal obesity, smoking, alcohol consumption, cardiovascular disease, atrial fibrillation, and lack of physical exercise. Hypertension was defined as either two consecutive blood pressure measurements with systolic pressure ≥140 mmHg or diastolic pressure ≥90 mmHg, or a prior diagnosis of hypertension, or current use of antihypertensive medication. Hyperglycemia was defined as fasting blood glucose ≥7.0 mmol/L, or a diagnosis of diabetes, or current use of hypoglycemic drugs. Hyperlipidemia was defined as total cholesterol ≥5.2 mmol/L, or triglycerides ≥1.7 mmol/L, or a diagnosis of hyperlipidemia, or current use of lipid-lowering medication. Smoking was defined as smoking at least one cigarette per day for more than six months. Alcohol consumption was defined as consuming ≥7 standard drinks per week for more than six months, with one standard drink corresponding to approximately 10 g of alcohol, equivalent to 300 mL of beer, 50–100 mL of liquor (Chinese Baijiu), or 100 mL of rice wine. Abdominal obesity was defined as a waist circumference >90 cm in men and >85 cm in women.

All participants underwent MRI scans in Zhongnan hospital, including 3D T1, 3D T2 fluid-attenuated inversion recovery (FLAIR) and DTI. A full set of neuropsychological assessments were conducted. Clinical information, including demographics, medical histories, lifestyles and results of basic laboratory tests, was collected. The diagnosis of VCI was determined by two experienced senior neurologists following the VICCCS guideline (1).

### 2.2 Neuropsychological assessments

All participants underwent comprehensive neuropsychological assessments conducted by an experienced neuropsychologist, including Montreal Cognitive Assessment (MoCA), Mini-Mental State Examination (MMSE), Trail-Making Test A (TMT-A), Trail-Making Test B (TMT-B), (Instrumental) Activities of Daily Living (ADL/IADL), Hamilton Anxiety Rating/Hamilton Depression Rating Scale (HAMA/HAMD), Boston Naming Test (BNT-15) or Verbal Fluency Test (VFT-3 min) and Chinese Auditory Verbal Learning Test (CAVLT). All researchers involved in the neuropsychological evaluations received standardized internal training prior to the initiation of the study.

### 2.3 Clinical information

We gathered demographic data, encompassing age, sex, body mass index (BMI), and educational level. Additionally, we compiled information on lifestyles and medical histories, such as smoking, exercising status, hypertension history, diabetes history, hyperlipidemia history, coronary heart disease (CHD), history of infarction, and intracranial vascular stenosis or occlusion history. Furthermore, we obtained results from various laboratory tests, including systolic blood pressure (SBP), diastolic blood pressure (DBP), fasting blood glucose (FBG), total cholesterol (TC), triglycerides (TG), low-density lipoproteins (LDL), and high-density lipoproteins (HDL).

### 2.4 Imaging features

**Figure 1:**
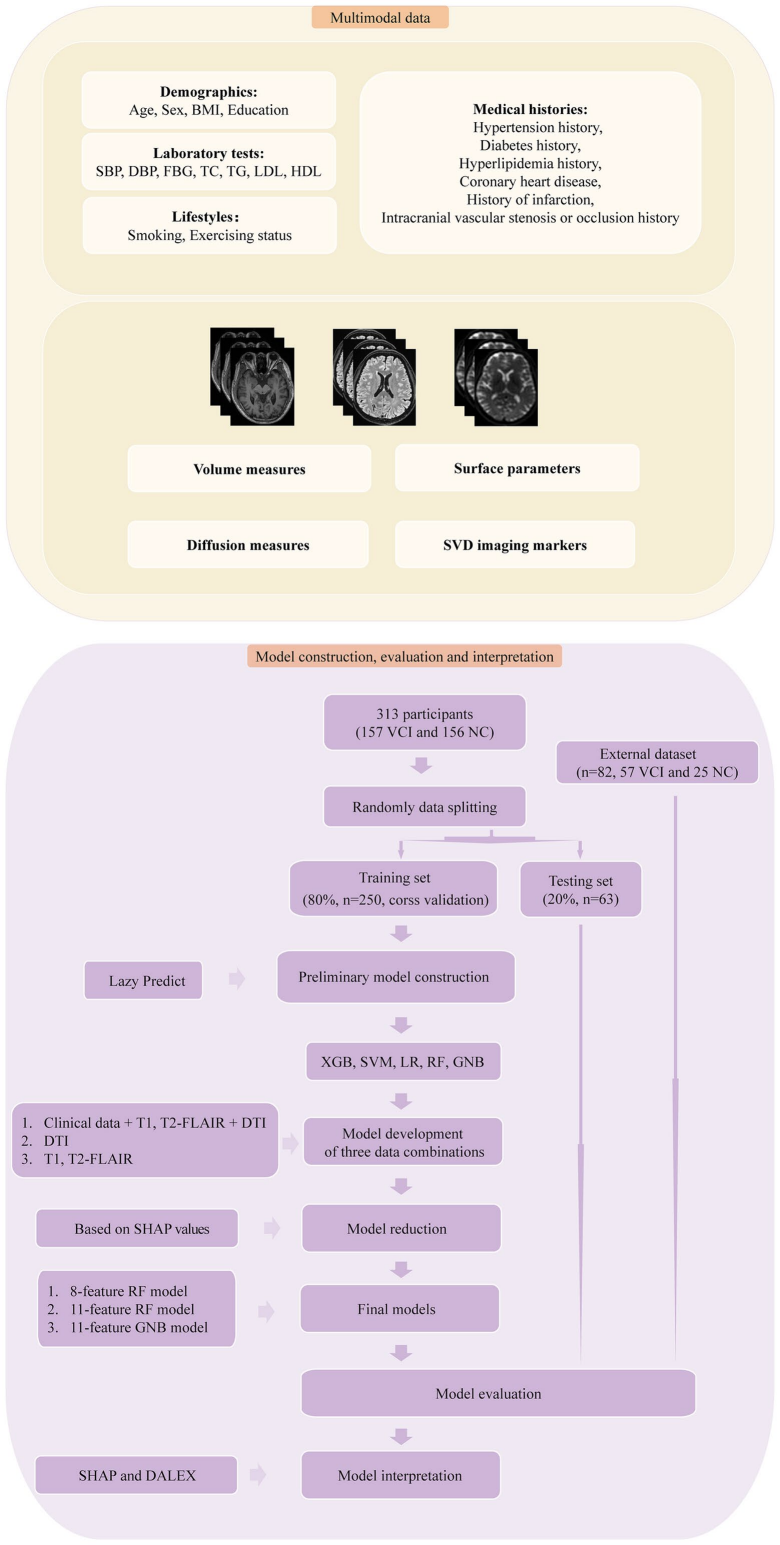
The framework for multimodal data processing, model construction, model evaluation and model interpretation. Multimodal data included various inputs, including clinical data and multimodal MRI. VBM, SBM and DTI measures were extracted from T1 and DTI, while SVD imaging markers were derived from T1 and T2-FLAIR. The models were developed using four ML algorithms and three combinations of data modalities. Model reduction was performed based on SHAP values. Subsequently, the final models underwent evaluation, external validation, and further interpretation.

Multimodalities MRI were collected, and measures from T1, T2-FLAIR and DTI were derived for subsequent ML modeling (Figure 1). T One major cause of VCI is SVD. In assessing the primary manifestations of SVD through imaging, we employed STRIVE criteria (25). Lacune, WMH and Enlarged perivascular spaces (EPVS) were assessed based on semi-quantified visual rating scale and fuzzy localizations. Voxel-based morphometry (VBM) and surface-based morphometry (SBM) were employed to T1 images to extract volume measures and surface parameters of various regions. Diffusion metrics of various ROIs were obtained from DTI, including fractional anisotropy (FA), mean diffusivity (MD), radial diffusivity (RD), and axial diffusivity (AD). The details of multimodalities MRI protocols and images processing are shown in Appendix S1.

### 2.5 Model construction and model reduction

For model construction, we utilized a comprehensive dataset that included demographics, medical histories, lifestyles, results of basic laboratory tests, SVD imaging markers, and processed MRI imaging measures (Figure 1). Our preliminary analysis involved conducting independent samples T-tests and Levene’s tests, followed by Student’s T-tests for data with homogeneity of variance and Welch’s tests for the rest. Imaging measures with a *p*-value less than 0.01 were selected (26, 27). The data underwent standardization through one-hot encoding and Z-score transformation independently across different combinations of data modalities. To refine the feature set, we applied the least absolute shrinkage and selection operator (LASSO), determining the penalty coefficient via 10-fold cross-validation (27). The dataset was randomly divided into training and testing sets in a ratio of 8:2, with the training set for model development and the testing set for subsequent internal validation. We used the Lazy Predict (0.2.12) to construct preliminary models and identify superior models for further development (28). Subsequently we employed five ML algorithms: Extreme Gradient Boosting (XGB), Support Vector Machine (SVM), Logistic Regression (LR), Gaussian Naive Bayes (GNB) and Random Forest (RF), constructing models based on diverse multimodal measures (clinical data, T1, T2-FLAIR, and DTI measures; T1 and T2-FLAIR measures; DTI measures). Model development included 10-fold cross-validation and grid search to determine the optimal hyperparameters. The best hyperparameters for the models were detailed in Appendix S1. Additionally, we sought to identify the minimum necessary variables for our ML models to enhance clinical applicability and simplify interpretation. Through the SHAP value ranking and a sequential forward selection strategy, we refined our models to those with stable area under the receiver operating curve (AUC) values that did not significantly change with the addition of more variables. Therefore, model reduction was carried out, resulting in the determination of the final models, which exhibited no significant changes in performance compared to the initial models. To address the issue of interpretability, we utilized SHAP (0.42.1) and moDel Agnostic Language for Exploration and eXplanation (DALEX 1.5.0) for further model analysis (21, 29, 30).

### 2.6 External validation

We conducted external validation using a dataset of 82 participants from the General Hospital of the Yangtze River Shipping and the Third People’s Hospital of Hubei Province, collected from 2022 to 2023. This dataset included 57 patients with VCI and 25 cognitive normal participants, adhering to the same inclusion and exclusion criteria as the Zhongnan VCI cohort.

### 2.7 Statistical analysis

Differences in demographics between patients with VCI and participants with normal cognitive function were examined using independent samples T-Test for normally distributed continuous variables, Mann–Whitney U test for non-normally distributed ones, and the chi-squared test for categorical variables. Normality was assessed through visual inspection and the Shapiro–Wilk test. Few missing values of laboratory results were imputed using the median, as detailed in the Appendix S1 (26, 31). Model performance was assessed using the receiver operating curve (ROC), the precision-recall (PR) curve and the average precision (AP). Additional model performance metrics, including accuracy, specificity, recall, precision, F1-score, and decision curve analysis (DCA), were also calculated. The 95% confidence intervals (CIs) for model evaluation metrics were generated with 1,000 bootstrap sets. The DeLong test was employed for ROC comparison. For ranking feature importance, SHAP utilized mean absolute SHAP values, while DALEX used a loss function of 1-AUC. Additionally, DALEX calculated variable importance through 10 permutations. Partial correlations were utilized to assess the associations between the selected imaging features of the final models and the scores of neuropsychological assessments, including MoCA, MMSE, TMT-A and TMT-B, independently in VCI and normal cognition group. Sex, age and education were controlled as covariates, and the Benjamini-Hochberg false discovery rate (FDR) was applied for multiple comparisons. Statistical significance was set at *p* < 0.05. DeLong test was performed using MedCalc software version 20.022, and other statistical analyses were conducted in IBM SPSS Statistics 27. The entire model construction process was implemented using the Scikit-learn package (1.2.2) in the Python environment.

## 3 Results

### 3.1 Participants and demographics

Our analysis included an internal dataset of 313 participants (Table 1) and an external dataset of 82 participants (Supplementary Table S1). The internal dataset comprised 157 participants with VCI and 156 cognitive normal participants with risk factors, which was used for model deviation and internal testing. The hold-out external dataset was utilized for external validation.

**Table 1:** Demographics.

Demographics	Derivation cohort number (%) or mean (SD, score range)		
	Patients with VCI	Participants with normal cognition	*p*
Age(years)	62.38 (6.62, 44–76)	59.93 (6.74, 48–75)	**<0.001**
Education (years)	10.83 (3.00, 6–19)	13.97 (3.19, 6–23)	**<0.001**
Sex, female, *n* (%)	47 (29.93%)	73 (46.79%)	**0.002**
BMI (kg/m^2^)	24.04 (2.53, 17.30–30.80)	24.64 (3.06, 18.29–38.06)	0.120
MMSE	24.48 (4.78, 6–30)	28.63(1.25, 25–30)	**<0.001**
MoCA	18.13 (4.85, 6–27)	25.53(2.28, 19–30)	**<0.001**
Smoke, *n* (%)	88 (56.05%)	54 (34.62%)	**<0.001**
Exercise, *n* (%)	117 (74.52%)	100 (64.10%)	**0.046**
Intracranial vascular stenosis or occlusion history, *n* (%)	80 (50.96%)	11 (7.05%)	**<0.001**
Infarct history, *n* (%)	102 (64.97%)	24 (15.38%)	**<0.001**
CHD, *n* (%)	18 (11.46%)	9 (5.13%)	0.073
Hypertension history, *n* (%)	117 (74.52%)	91 (58.33%)	**0.002**
Diabetes history, *n* (%)	64 (40.76%)	58 (37.18%)	0.516
Hyperlipidemia history, *n* (%)	58 (36.94%)	101 (64.74%)	**<0.001**
SBP	136.09 (14.60, 100–180)	129.90 (16.27, 92–170)	**<0.001**
DBP	79.76 (10.63, 58–124)	79.94(11.30,53–111)	0.787
FBG (mmol/L)	6.20 (2.76, 3.05–28.53)	5.73 (1.45, 2.81–11.36)	0.068
TC (mmol/L)	4.11 (1.01, 2.12–7.69)	4.60 (0.90, 2.51–7.04)	**<0.001**
TG (mmol/L)	1.68 (1.20, 0.33–11.69)	1.75 (1.24, 0.38–10.01)	0.607
HDL (mmol/L)	1.08 (0.34, 0.52–3.16)	1.28 (0.35, 0.70–3.16)	**<0.001**
LDL (mmol/L)	2.42 (0.79, 0.99–5.10)	2.69 (0.76, 1.08–4.85)	**0.002**
CSO-EPVS	0.30 (0.49, 0–2)	0.13 (0.41, 0–3)	**<0.001**
BG-EPVS	1.10 (0.30, 1–2)	1.00 (0.16, 0–2)	**0.001**
Deep lacune	0.39 (0.78, 0–5)	0.18 (0.51, 0–3)	**<0.001**
Lobar lacune	2.97 (4.43, 0–23)	0.38 (1.52, 0–15)	**<0.001**
Fazekas-PV	1.93 (1.00, 0–3)	0.87 (0.84, 0–3)	**<0.001**
Fazekas-DEEP	1.64 (1.02, 0–3)	0.80 (0.75, 0–3)	**<0.001**

BMI, body mass index; CHD, coronary heart disease; SBP, systolic blood pressure; DBP, diastolic blood pressure; FBG, fasting blood glucose; TC, total cholesterol; TG, triglycerides; LDL, low-density lipoproteins; HDL, high-density lipoproteins; Fazekas-PV, Fazekas scale in periventricular white matter; EPVS, enlarged perivascular spaces; BG, basal ganglia; CSO, centrum semiovale. Bold values represent statistical significance at the *p*<0.05 level.

### 3.2 Model performance, model reduction, and identification of final model

**Figure 2:**
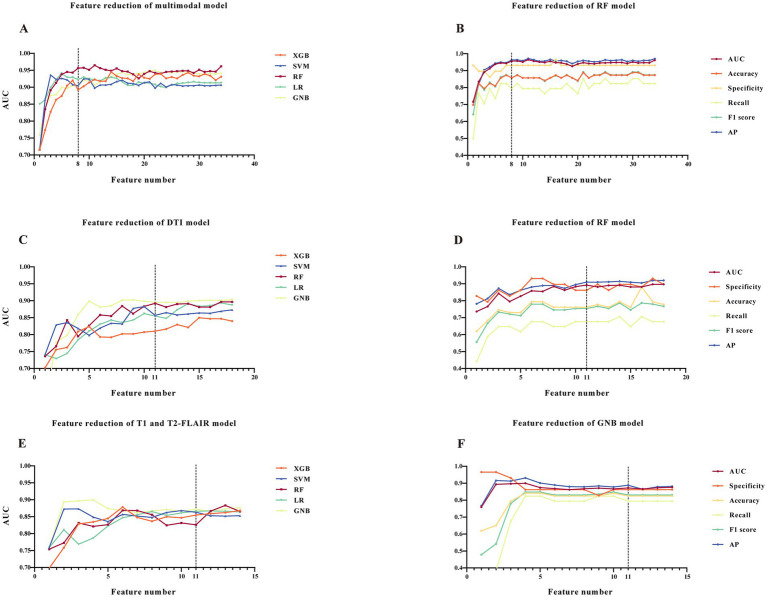
Model reduction and determination of final models. **(A,C,E)** AUCs with varied number of features of five ML models. **(B,D,F)** AUC, accuracy, specificity, recall, F1 score and AP with varied number of features of the determined final models. **(A,B)** Models constructed with clinical data, T1, T2-FLAIR and DTI measures. **(C,D)** Models of DTI measures. **(E,F)** Models of T1 and T2-FLAIR measures. The dotted line represents the decision point of the final models. Abbreviations: ROC, receiver-operating-characteristic; AUC, area under the ROC curve; AP, average precision; ML, machine learning; RF, random forest; XGB, eXtreme gradient boosting; SVM, support vector machine; LR, logistic regression; GNB, gaussian naive bayes.

**Figure 3:**
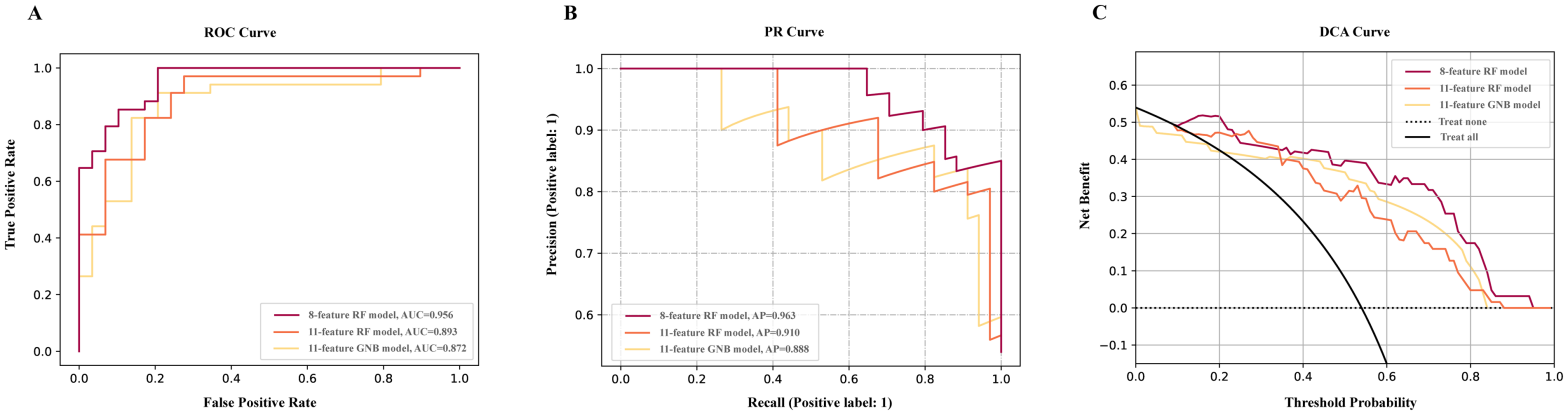
Performance of final models. The ROC curve **(A)** PR curve **(B)**, and DCA curve **(C)** of final models. 8-feature RF model constructed with clinical data, T1, T2-FLAIR and DTI measures. 11-feature RF model constructed with DTI measures. 11-feature GNB model constructed with T1 and T2-FLAIR measures.

Following feature selection, the initial model, which integrated clinical data along with T1, T2-FLAIR, and DTI measures, incorporated 34 variables. Additionally, the initial models exclusively using T1 and T2-FLAIR, and DTI measures contained 14 and 18 variables, respectively (Supplementary Figures S1–S3). The results of Lazy Predict demonstrated that RF model outperformed other models (Supplementary Table S2). For the comprehensive model utilizing clinical data and multimodal MRI (Supplementary Table S3), the RF model has the highest AUC (0.962) and AP value (0.970). Performance of the initial DTI model (Supplementary Table S4) and the model combining T1 and T2-FLAIR (Supplementary Table S5) measures was found to be lower than the comprehensive multimodal model. Adding more data modalities improved model performance. For DTI-specific models, the GNB model exhibited the highest AUC (0.904) and AP (0.922), followed by the RF model, which showed a slightly decreased AUC (0.897) and AP (0.920). The F1-score of the RF and GNB model was 0.767 and 0.640, respectively. Among models built with T1 and T2-FLAIR measures, the GNB model had the highest AUC (0.876). The model reduction process, guided by the ranking of SHAP values, demonstrated that after a certain point, as the number of features increased, the model’s performance trends stabilized (Figure 2). This observation led to the determination of a cut-off point for model reduction and the selection of key features. Combining the model performance metrics and model reduction process, the final models were ultimately determined. For models combining clinical data with measures from T1, T2-FLAIR, and DTI, an 8-feature RF model was selected. This model, with an AUC of 0.956, performed comparably to the initial 34-feature model (AUC = 0.962) without a significant difference (ΔAUC = 0.005, *p* = 0.725), while significantly reducing the number of variables required. It also achieved a high AP of 0.963, emphasizing the model’s clinical utility through its high performance with fewer variables (Figure 3). For models based on DTI measures, the 11-feature RF model was chosen, achieving an AUC of 0.892 and an AP of 0.910, without a significant change in classification capability (ΔAUC = 0.004, *p* = 0.698) from the 18-feature RF model before model reduction. For models utilizing T1 and T2-FLAIR measures, the 11-feature GNB model was identified, with an AUC of 0.872 and an AP of 0.888. This model showed no significant decrease in performance (ΔAUC = 0.004, *p* = 0.783) compared to the 14-feature GNB model (Table 2). The comparison of ROC, PR, and DCA curves between the initial models and the final models after model reduction showed no significant decline in performance (Supplementary Figure S4). In summary, the 8-feature RF model, the 11-feature RF model, and the 11-feature GNB model were identified as the final models for three distinct data modality combinations.

**Table 2:** Performance of the final models with different modalities.

Modalities	Clinical data + T1 and T2-FLAIR + DTI	DTI	T1 and T2-FLAIR
Algorithm	RF	RF	GNB
Number of features	8	11	11
ΔAUC and *p*	ΔAUC = 0.005 *p* = 0.725	ΔAUC = 0.004 *p* = 0.698	ΔAUC = 0.004 *p* = 0.783
AUC	0.956 [0.919–0.988]	0.892 [0.813–0.954]	0.872 [0.793–0.945]
AP	0.963 [0.929–0.990]	0.910 [0.836–0.964]	0.888 [0.809–0.959]
F1-score	0.857 [0.769–0.930]	0.754 [0.643–0.842]	0.831 [0.735–0.909]
Accuracy	0.857 [0.778–0.937]	0.762 [0.667–0.841]	0.825 [0.746–0.905]
Precision	0.931 [0.846–1.0]	0.852 [0.741–0.960]	0.871 [0.769–0.967]
Recall	0.794 [0.677–0.909]	0.676 [0.546–0.807]	0.794 [0.676–0.906]
Specificity	0.931 [0.846–1.0]	0.862 [0.758–0.963]	0.862 [0.750–0.963]

ΔAUC and *p*: the difference of AUC and significance level between the final models and the initial models without model reduction. AUC, area under the ROC curve; AP, average precision; RF, random forest; GNB, gaussian naive bayes. The AUC was calculate using the Scikit-learn package (1.2.2); the DeLong test was performed using MedCalc software version 20.022; the 95% confidence intervals (CIs) were estimated using 1,000 bootstrap resamples.

In DCA (Figure 3C) of final models, with a threshold approximately 0.1 to 0.24 and over 0.34, the net benefit of the multimodal model was the largest. The DCA curve revealed that the multimodal model had a wider range of threshold probability and a higher net benefit, thence, had greater clinical utility.

The DeLong test showed a statistically significant difference between the final model incorporating only T1 and T2-FLAIR features and the comprehensive final model that included clinical data, T1 and T2-FLAIR, and DTI. The difference in ROC areas was 0.084, with a standard error of 0.033, a z statistic of 2.524, 95% CIs ranging from 0.019 to 0.150, and a *p*-value of 0.012. This outcome suggests that the imaging indicators derived from T1 and T2-FLAIR alone are insufficient for a comprehensive assessment of VCI. No significant differences were observed among the other final models.

### 3.3 External validation of the final model

For external validation, the final models were tested on external dataset. The 8-feature RF model, which incorporates clinical data and multimodal MRIs, achieved an AUC of 0.919 (Supplementary Table S6). This performance was comparable to that observed in the internal validation dataset, with no significant decrease in performance (ΔAUC = 0.038, *p* = 0.308). The AP of this final model on external dataset was 0.966, respectively. For the 11-feature RF model specific to DTI measures, the AUC was 0.779, slightly lower but not significantly different (ΔAUC = 0.114, *p* = 0.108) from the internal validation performance. In contrast, the 11-feature GNB model for T1 and T2-FLAIR measures demonstrated an AUC significantly lower (ΔAUC = 0.301, *p* < 0.001) than its performance in internal validation.

### 3.4 Model interpretation

**Figure 4:**
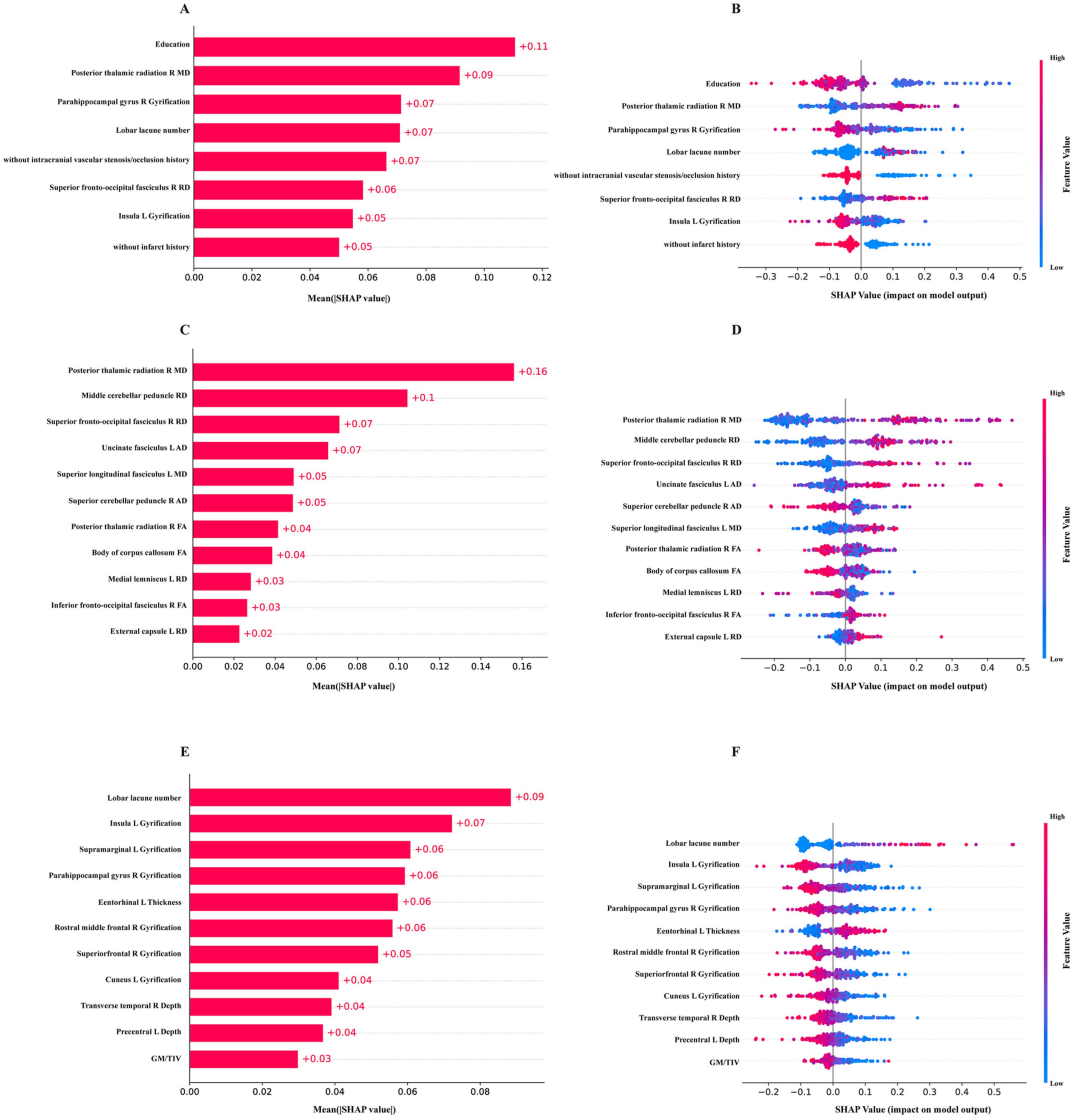
The SHAP values of the final models. **(A,B)** the mean absolute SHAP values and the distribution of SHAP values for the 8-feature RF model constructed with clinical data, T1, T2-FLAIR and DTI measures. **(C,D)** SHAP values for the 11-feature RF model of DTI measures. **(E,F)** SHAP values for the 11-feature GNB model of T1, T2-FLAIR measures.

The final 8-feature RF model demonstrated reliable and strong performance in both internal and external validations. Consequently, we utilized SHAP and DALEX for further interpretation of this model and to assess the contribution of the input variables. For SHAP, the variable contributions were ranked and displayed based on their mean absolute SHAP values (Figure 4A). A SHAP value greater than zero indicates a contribution towards a positive class prediction, in this case, indicating cognitive impairment (Figure 4B). For DALEX, Education level and the MD of right posterior thalamic radiation (PTR) emerged as the most significant features. These were followed by the gyrification of the right parahippocampal gyrus, the count of lobar lacunes, history of intracranial vascular stenosis or occlusion, RD of the right superior fronto-occipital fasciculus, gyrification of the left insula, and infarct history. Imaging features played a pivotal role, and their importance was consistent across both the 11-feature RF model and the 11-feature GNB model, which were solely based on imaging measures (Figures 4C–F). This consistency across models indicates that our approach is stable to variations in architecture and input, rather than relying on a limited set of clinical features for decision-making. For the 8-feature RF model, both DALEX and SHAP yield the same importance ranking (Supplementary Figure S5a), affirming the reliability of the interpretations. In the case of the 11-feature RF model, most of the importance rankings align, with both methods identifying the same top four features (Supplementary Figure S5b). The importance ranking of the 11-GNB model is not consistent, but both analyses concur on the most important feature (Supplementary Figure S5c). SHAP force plots help interpret how each feature of a model influences a specific prediction relative to a baseline. For each of the three final models, one VCI prediction and one NC prediction were selected and are presented in Supplementary Figure S6 for further explanation.

### 3.5 Neuropsychological assessments

We evaluated the association between the key imaging features identified by SHAP and DALEX and neuropsychological assessments within the VCI group. Significant associations were observed, further supporting the critical imaging markers identified by our models (Supplementary Table S7).

## 4 Discussion

We gathered data from a comprehensive VCI cohort, which included clinical information, neuropsychological assessments, and multimodal MRI sequences. By employing ML algorithms, we constructed models with a variety of data inputs. Among various data combinations tested, our results showed that the model incorporating all data modalities, including clinical data, T1, T2-FLAIR, and DTI measures, achieved the most favorable performance. The model’s performance was enhanced by the inclusion of additional data modalities. Furthermore, the DTI-based model outperformed than the model constructed with T1 and T2-FLAIR measures, demonstrating superior classification ability. To enhance usability, we employed model reduction based on SHAP values to rank the importance of variables. Additionally, DALEX was implemented to further validate these variables, affirming the reliability of the interpretations (30). Our best performing final model, which includes all data modalities with only eight input variables, exhibited no significant decline in performance compared to the initial model with 34 features. During external validation, the final model constructed with all modalities and the DTI-based final model exhibited stable performance, showing no significant difference compared to internal validation. This demonstrates the model’s reliability and good clinical applicability across diverse sample sets.

VCI has a complex etiology and diverse pathogenesis, and its precise pathogenesis has not yet been fully elucidated (5, 32). In actual practice, different individuals may exhibit varied symptoms and disease trajectories, and the mechanisms of interaction among various cognitive impairment risk factors, as well as their relative contributions, remain difficult to define accurately (22). Identifying new imaging markers and consolidating existing ones, as well as expanding the use of neuroimaging techniques and developing new models, is crucial for achieving an early and accurate diagnosis of VCI (33). The application of advanced multimodal neuroimaging techniques and machine learning models enables the early evaluation and identification of individuals at risk of VCI (33, 34). DTI is a sensitive MRI approach in detecting microstructural damage, capable of capturing subtle WM alterations (35). Prior study suggested that DTI measures may be more decisive markers for detecting cognitive deficits compared to conventional imaging markers (10). In our study, the DTI model showed stable performance in an external dataset, showing no significant decline of accuracy. However, a significant performance decline was observed in the external validation for the model based on T1 and T2-FLAIR measures. This decline could be attributed to demographic differences between the internal and external datasets, the patients with VCI in the external dataset typically had milder symptoms and were likely in an earlier stage of VCI. Microstructural damages are more common in VCI, yet conventional MRI techniques often demonstrate limited sensitivity to these changes (23). Conversely, prior research has shown that DTI measures are capable of detecting subtle alterations in WM regions, even during the preclinical stage of VCI (36). Aligning with existing studies, our research further confirms the sensitivity and applicability of DTI across a range of clinical stages within the VCI population.

Given the diverse and complex pathophysiology and risk factors associated with VCI, the integration of multimodal data and the adoption of novel and quantitative imaging approaches improve diagnostic specificity and sensitivity. However, few models have been developed for the diagnosis and prediction of VCI, and they were all based on small sample studies (19, 26). Our approach using ML to integrate clinical and multimodal MRI data for VCI diagnosis was developed on a relatively larger dataset and included both internal and external validation. The reduction of features in these models further improved their clinical usability. Moreover, advancements in computing power and processing capabilities make ML a promising approach to addressing clinical challenges (37). In our study, the RF model showed superior performance, attributed to their capability to handle complex and nonlinear data commonly encountered in medical scenarios (38, 39).

According to SHAP and DALEX analysis, our study confirmed the importance of several imaging markers, especially those derived from DTI. These markers demonstrated a meaningful association with cognitive functions, such as attention, processing speed, executive function, and global cognition. The influential cortical regions identified in our study encompass the frontal and temporal lobes, parahippocampal gyrus, cuneus, precuneus, supramarginal gyrus, precentral gyrus, and insula. Consistent with prior research, the gyrification of these areas was significantly linked to cognitive functions (40, 41). Regarding DTI measures, the PTR, cerebellar peduncle, superior fronto-occipital fasciculus, uncinate fasciculus and the body of corpus callosum were identified as the five most critical WM regions. Alterations in the PTR are found to associate with executive function, a conclusion supported by our findings that linked decreased FA of the PTR with extended completion times on the TMT-A and TMT-B (42). Additionally, cognitive decline has been associated with dysfunction in the cortical-cerebellar-cortical loop, notably the middle cerebellar peduncle, the largest of the three peduncles (43, 44). Our study found significant associations between higher RD of the middle cerebellar peduncle and longer completion times for TMT-B. The corpus callosum, which comprises WM commissural fibers connecting the left and right hemispheres, plays a vital role in cognitive functions. SHAP analysis indicated that an increase in the FA of the body of corpus callosum had a positive impact on cognition, supporting previous research and suggesting its utility as an independent indicator of VCI (45). Additionally, the majority of DTI indicators in the model were aligned with the lateral cholinergic pathway. The dysfunction of the cholinergic pathway and the cortical disconnection hypothesis are increasingly recognized for their roles in the manifestation and progression of VCI (24). Previous study using diffusion MRI with tractography to isolate lateral cholinergic tracts have found correlations between diffusion measures of these tracts and cognitive functions (46). In our study, the correlation between cognitive outcomes and diffusion measures along lateral cholinergic tracts may support the hypothesis that lesions in these segments disrupt the integrative function of WM microstructure, which is then reflected in cognitive performance (47). Restoring the cholinergic system through the use of cholinesterase inhibitors represents one of the primary therapeutic approaches for treating cognitive impairment in VCI and AD (24, 48–50). However, their effectiveness in the early stage of mild cognitive impairment remains controversial (51). Studies have indicated that the therapeutic effect of cholinergic treatment depends significantly on cholinergic integrity in the early stages (51). Monitoring these DTI biomarkers *in vivo* may provide objective indicators reflecting the integrity of cholinergic pathways, enabling the stratification of patients who would specifically benefit from cholinergic-oriented interventions (49, 51). Furthermore, these markers could serve as indicators for evaluating therapeutic effects, potentially before clinical cognitive improvements become apparent. These insights might advocate for the consideration of cholinergic therapies for VCI, with diffusion abnormalities in particular regions acting as sensitive markers for early detection and intervention to prevent or delay cognitive decline (52). Overall, when incorporated into a ML model, these imaging indicators derived from multimodal MRI demonstrate potential in assessing VCI status and show significant correlations with cognitive decline in VCI patients.

Additionally, several clinical factors, including education, HDL, SBP levels, and specific medical histories, also contributed to the diagnosis of VCI. Notably, a history of hyperlipidemia was identified as a protective factor in our models, possibly due to the beneficial effects of statins that target atherosclerotic factors crucial in reducing vascular-related cognitive decline (53). Recent studies have identified links between cardiovascular risk factors and structural brain changes, emphasizing the importance of managing these factors to promote healthy brain aging (54). Screening and addressing modifiable risk factors, such as educational level and vascular health, could strengthen brain reserve and potentially prevent or delay the onset of dementia (11, 55, 56).

Our study encountered several limitations. Primarily, our participants were predominantly from central China, and the external validation dataset was relatively small, limiting the model’s applicability in other regions. Nevertheless, it’s important to highlight that our dataset originated from multiple centers, and the model underwent external validation. The inherent heterogeneity within our dataset provides preliminary clinical evidence supporting its potential for broader application and evaluation across diverse regions. Nonetheless, further validations across multiple regions and longitudinal studies are crucial to bolster the robustness of our findings. Secondly, our cohort exhibited a higher level of education and participants unable to complete neuropsychological assessments were excluded, possibly skewing the generalizability of our results. Thirdly, the inclusion period of our study encompasses the dates of the COVID-19 pandemic. Although all participants tested negative for the virus via nucleic acid tests at recruitment, 16 participants recruited in 2023 reported a history of infection, which could potentially influence the DTI data (57). We are currently recruiting more participants and conducting follow-up studies to overcome these limitations. Future research endeavors should aim to incorporate a wider array of MRI modalities and more advanced algorithms such as federated learning to accumulate multicenter big data while addressing data privacy concerns, thereby enabling a more accurate delineation of early VCI changes and the development of novel intervention strategies (58).

In summary, our study developed ML models capable of distinguishing between cognitive normal individuals at risk and those with VCI, achieving increased clinical applicability through the use of fewer input variables. By integrating clinical data and multimodal MRI, the models demonstrated not only enhanced precision in identifying patients with VCI but also generalization ability in external validation. We also highlighted the importance of DTI, perhaps more important than conventional imaging markers. Our research has uncovered several potential imaging markers sensitive to the detection of VCI, highlighting the critical role of specific cortical and WM abnormalities in clarifying the manifestations of VCI.

## Data Availability

The data analyzed in this study will be made available on reasonable request to the corresponding author. Requests to access the anonymized datasets should be directed to zhangjj@whu.edu.cn.
