# Supplementary material for: Evaluation of vascular cognitive impairment and identification of imaging markers using machine learning: a multimodal MRI study
Source: Front Neurol. 2025 May 29;16:1505739. doi: 10.3389/fneur.2025.1505739 (PMC12158719; doi:10.3389/fneur.2025.1505739)
Supplement: Supplementary file 1 [file Supplementary_file_1.docx]

**Supplementary material：Appendix S1**

1. **Supplementary Methods**
   1. MRI protocols
   2. Images (T1, T2-FLAIR and DTI) processing
   3. Best hyperparameters for the final models
   4. Details of missing values
2. **Supplementary Figures**
   1. Figure S1: SHAP values of the initial 34-feature RF model (All data modalities)
   2. Figure S2: SHAP values of the initial 18-feature RF model (DTI)
   3. Figure S3: SHAP values of the initial 14-feature GNB model (T1 & T2-FLAIR)
   4. Figure S4: Performance comparison of final models after feature reduction
   5. Figure S5: Feature importance of final models determined using DALEX
   6. Figure S6: SHAP Force Plots for Three Final Models Showcasing VCI and NC Example Predictions
3. **Supplementary Tables**
   1. Table S1: Demographics of external dataset
   2. Table S2: Top 10 performing models identified by Lazy Predict
   3. Table S3: Performance comparison of initial 34-feature models constructed with different ML algorithms (All data modalities)
   4. Table S4: Performance comparison of initial 18-feature models constructed with different ML algorithms (DTI)
   5. Table S5: Performance comparison of initial 14-feature models constructed with different ML algorithms (T1 & T2-FLAIR)
   6. Table S6: Performance of the final models in external dataset
   7. Table S7: Correlations between discriminative imaging measures and neuropsychological assessments within VCI group
4. **Reference**
5. **Supplementary Methods**
   1. **MRI protocols**

Multiple modalities of MRI scans were acquired including high resolution 3D T1 images, 3D T2 FLAIR and DTI using a 3.0T SIMENS Trio. T1 images were acquired with a 3D MPRAGE sequence, the parameters were: repetition time (TR) = 6.7 s, echo time (TE) = 2.26 ms, flip angle = 9°, voxel size = 1×1×1 mm^3^, and 176 sagittal slices. 3D T2-FLAIR images were acquired using an inversion recovery MATRIX sequence with parameters: TR = 6000 ms, TE = 388 ms, time inversion (TI) = 2200 ms, echo train length = 141, bandwidth = 781 Hz/pixel, voxel size = 0.49×0.49×1 mm^3^, and 160 sagittal slices. DTI was acquired with an echo planar imaging sequence, the parameters were: TR = 9,200 ms, TE = 86 ms, field of view = 256 × 256 mm^2^, voxel size = 2 × 2 × 2 mm^3^, diffusion direction = 64.

- 1. **Images (T1, T2-FLAIR and DTI) processing**

One major cause of VCI is small vessel disease. In assessing the primary manifestations of small vessel disease (SVD) through imaging, we employed STRIVE criteria. Lacunes were quantified based on numbers observed in deep regions (basal ganglia, thalamus, caudate, internal, and external capsule) and lobar regions (centrum semiovale, frontal, parietal, insular, temporal, and occipital lobes). WMH were evaluated using the Fazekas scale in periventricular (PV) and deep white matter. Enlarged perivascular spaces (EPVS) were measured in the basal ganglia (BG) and centrum semiovale (CSO) using a scale ranging from 0 to 4 (0 = no EPVS, 1 = < 10 EPVS, 2 = 10–20 EPVS, 3 = 21–40 EPVS, and 4 = > 40 EPVS).

Voxel-based morphometry (VBM) was performed to assess volumetric changes using cat12, an additional toolbox based on spm12. The default pipeline of CAT12 was utilized for preprocessing, and the preprocessed T1 images underwent further segmentation^1, 2^. For spatial normalization, an optimized shooting approach was employed to register the T1 images from individual space to the MNI standard space. Total intracranial volume, gray matter, white matter, and cerebrospinal fluid volume were estimated and based on Anatomical Automatic labeling (AAL) Atlas, the volumes of various regions of interests (ROIs) were further extracted^3^.

Concurrently with the VBM segment step, we conducted surface-based morphometry (SBM), estimating cortical thickness and the central cortical surface through projection-based thickness. The surface reconstruction process included topological correction, aspherical mapping and spherical registration^4, 5^. A smoothing kernel of 15mm full width at half maximum Gaussian was used for thickness data, and 20mm for gyrification and fractal dimension data. Surface parameters, including cortical thickness, sulcus depth, gyrification and fractal dimension were then extracted based on the Desikan–Killiany (DK40) cortical Atlas for further analyze^6-8^.

DTI images were processed using the pipeline for analyzing brain diffusion images (PANDA 1.31) to execute the extraction of diffusion measures^9^. Regarding preprocessing, eddy current correction was applied to DTI images, while brain extraction for T1 images was conducted using Deep-brain (https://github.com/iitzco/deepbrain). Diffusion measures of various ROIs, including fractional anisotropy (FA), mean diffusivity (MD), radial diffusivity (RD), and axial diffusivity (AD), were extracted using the white matter atlas ICBM-DTI-81-FMRIB58^10^.

In summary, SVD imaging markers, volume and surface measures of various ROIs extracted from T1 image, and diffusion metrics derived from DTI were processed and utilized for subsequent model construction.

- 1. **Best hyperparameters for the final models**

The hyperparameters for the 8-feature RF model are: max_depth=4, n_estimators=120, and random_state=0. The hyperparameters for the 11-feature RF model are: max_depth=4, n_estimators=90, random_state=0. The hyperparameter for the 11-feature GNB model is: var_smoothing=1e-09.

- 1. **Details of missing values**

There were few missing values in demographics: 14 individuals lack BMI; 8 individuals simultaneously lack FBG, TC, TG, LDL, and HDL; 1 individual simultaneously lacks TC, TG, LDL, and HDL.

Abbreviations: BMI, body mass index; FBG, fasting blood glucose; TC, total cholesterol; TG, triglycerides; LDL, low-density lipoproteins; HDL, high-density lipoproteins.

1. **Supplementary Figures**
   1. **Figure S1 The SHAP values of the initial 34-feature RF model (All data modalities)**

a, the distribution of SHAP values; b, the mean absolute SHAP values. The initial 34-feature RF model was constructed with clinical data, T1, T2-FLAIR and DTI measures.


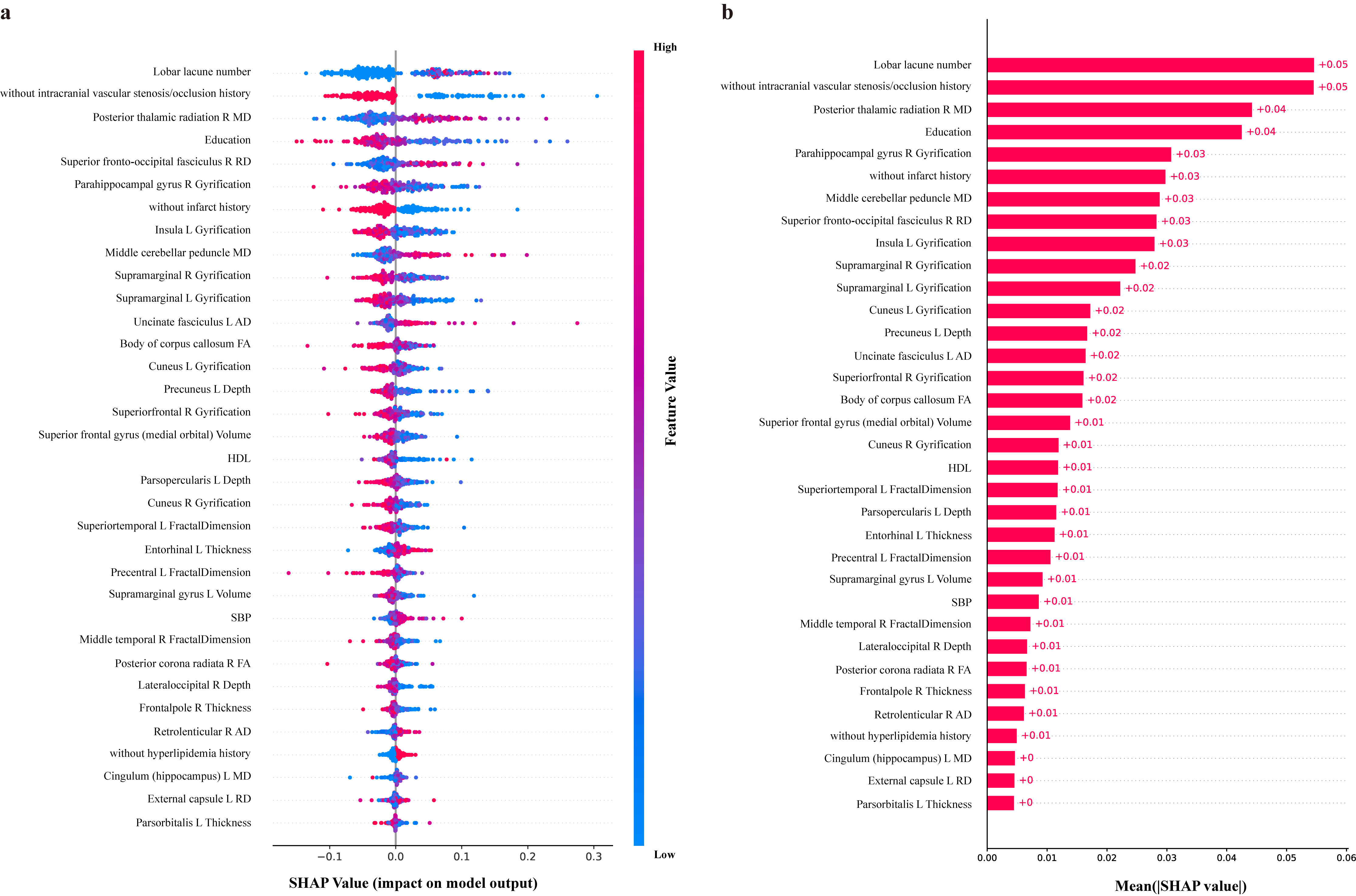


- 1. **Figure S2 The SHAP values of the initial 18-feature RF model (DTI)**

a, the distribution of SHAP values; b, the mean absolute SHAP values. The initial 18-feature RF model was constructed with DTI measures.


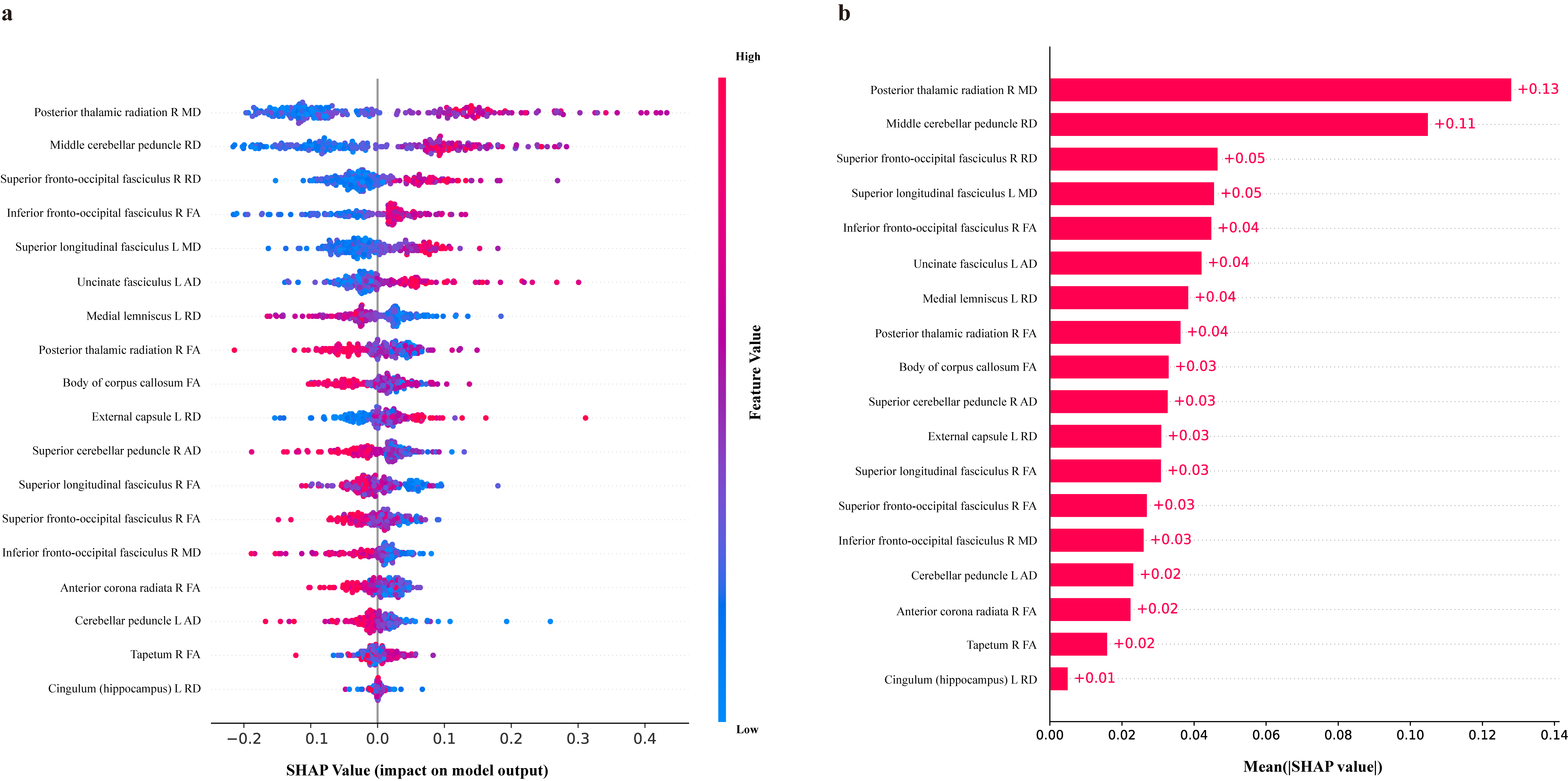


- 1. **Figure S3 The SHAP values of the initial 14-feature GNB model (T1 & T2-FLAIR)**

a, the distribution of SHAP values; b, the mean absolute SHAP values. The initial 14-feature GNB model was constructed with T1 and T2-FLAIR measures.


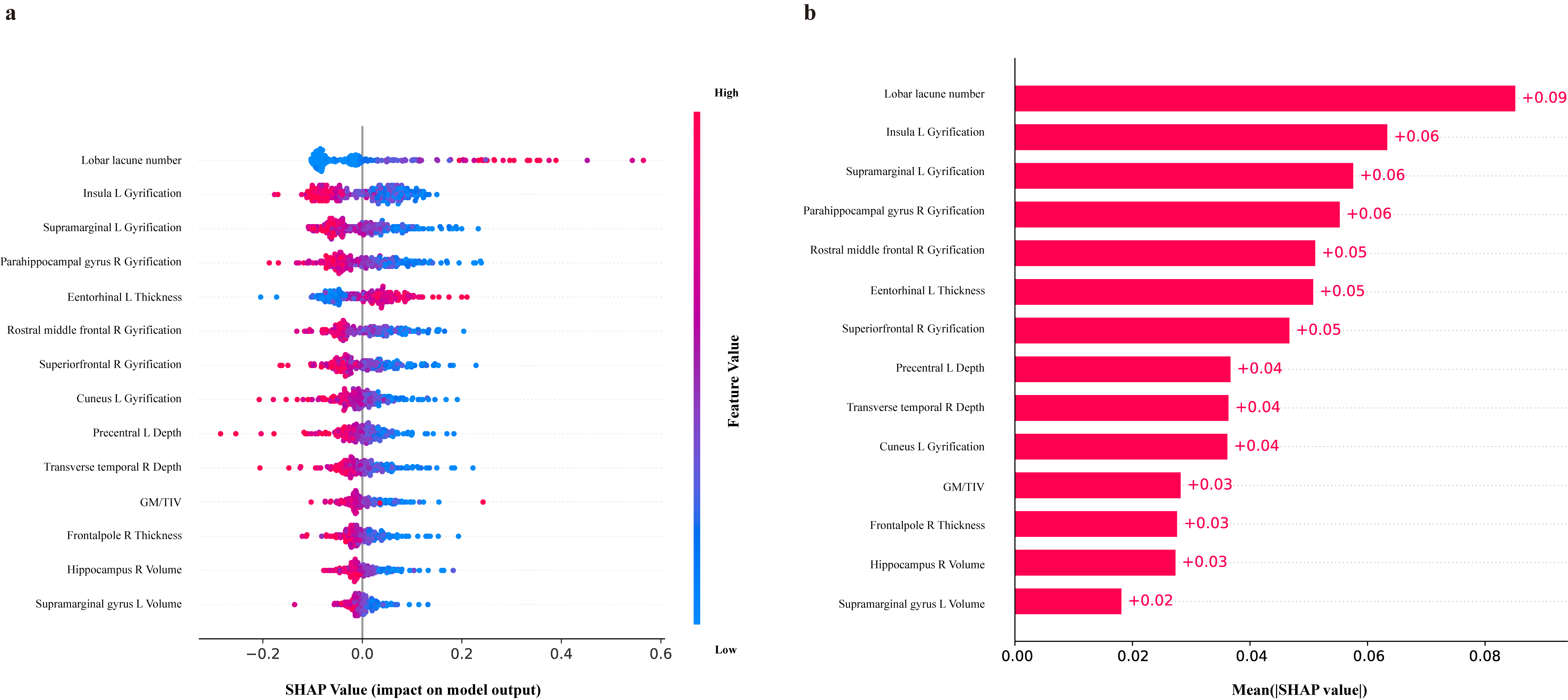


- 1. **Figure S4** **Performance comparison of initial models and final models after model reduction**

a, ROC for both the initial models and the final models, after model reduction, of the RF all-modalities model, the RF DTI model, and the GNB T1 and T2-FLAIR model

b, PR curves for both the initial models and the final models, after model reduction, of the RF all-modalities model, the RF DTI model, and the GNB T1 and T2-FLAIR model

c, DCA curves for both the initial models and the final models, after model reduction, of the RF all-modalities model, the RF DTI model, and the GNB T1 and T2-FLAIR model


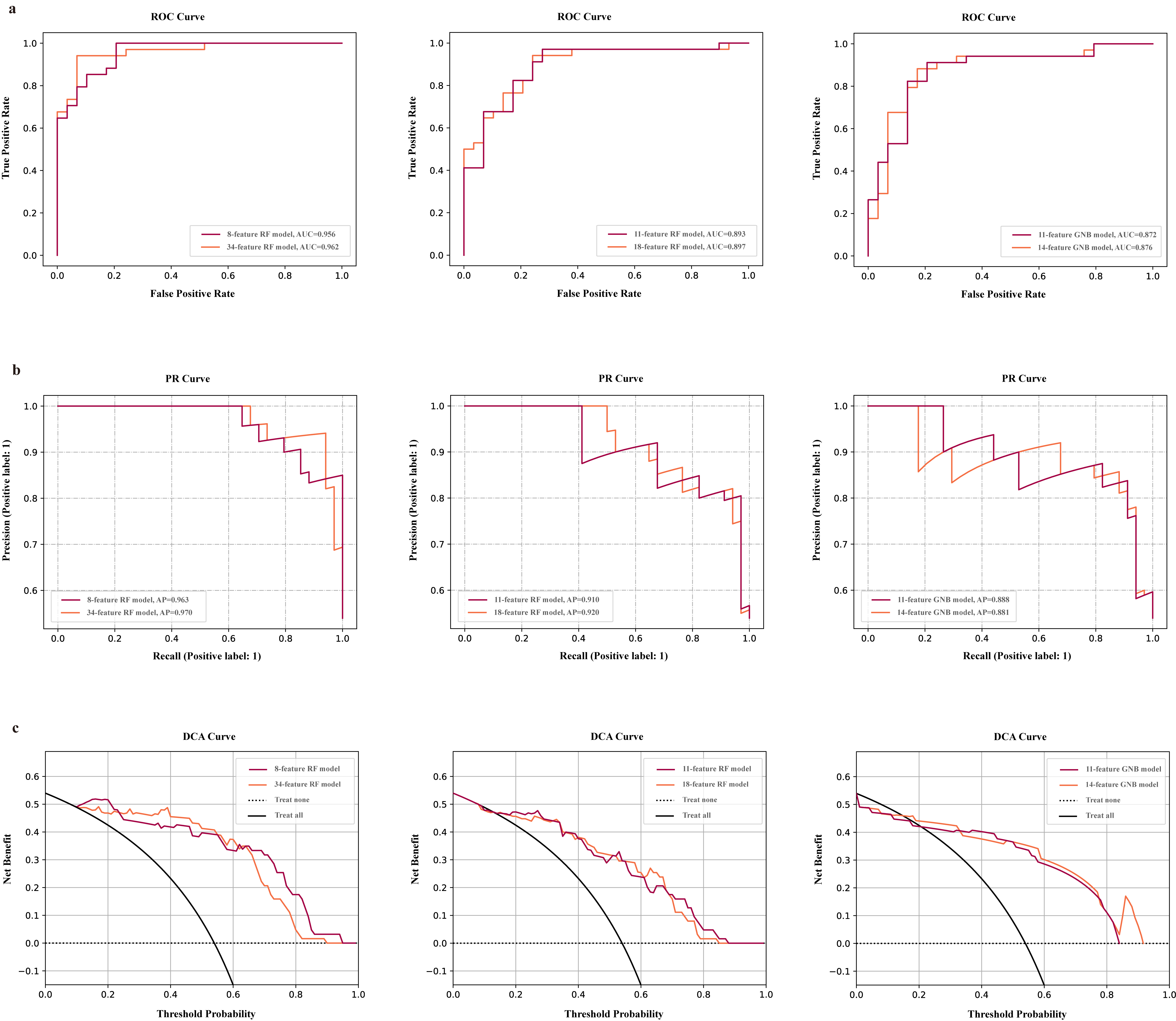


- 1. **Figure S5: Feature importance of final models determined using DALEX**

a, the feature importance of the final 8-feature RF model constructed with clinical data, T1, T2-FLAIR and DTI measures; b, the feature importance of the final 11-feature RF model of DTI measures; c, the feature importance of the final 11-feature GNB model of T1, T2-FLAIR measures.


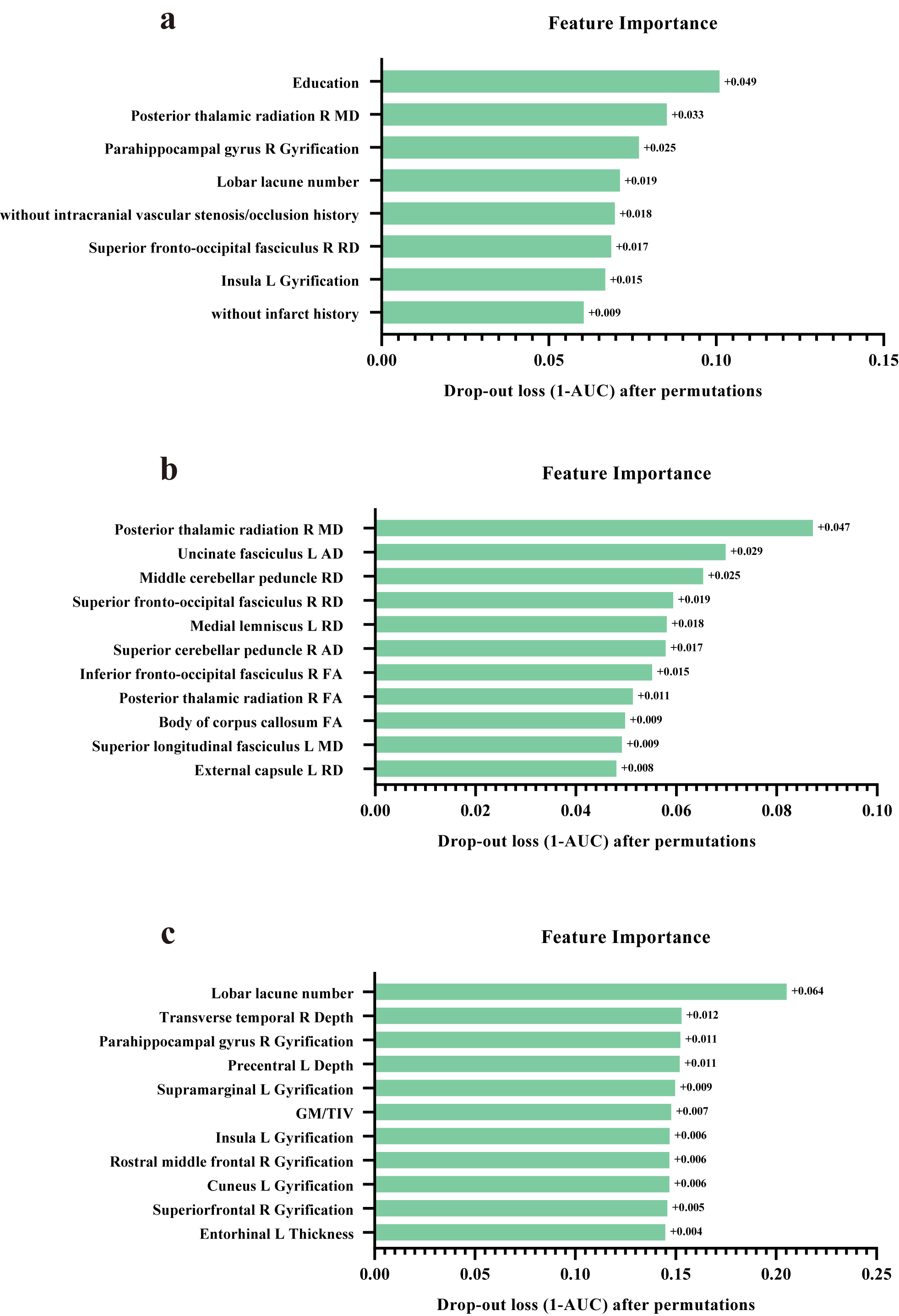


- 1. **Figure S6: SHAP Force Plots for Three Final Models Showcasing VCI and NC Example Predictions**

a, the VCI prediction of the final 8-feature RF model; b, the NC prediction of the final 8-feature RF model; c, the VCI prediction of the final 11-feature RF model; d, the NC prediction of the final 11-feature RF model; e, the VCI prediction of the final 11-feature GNB model; f, the NC prediction of the final 11-feature GNB model.


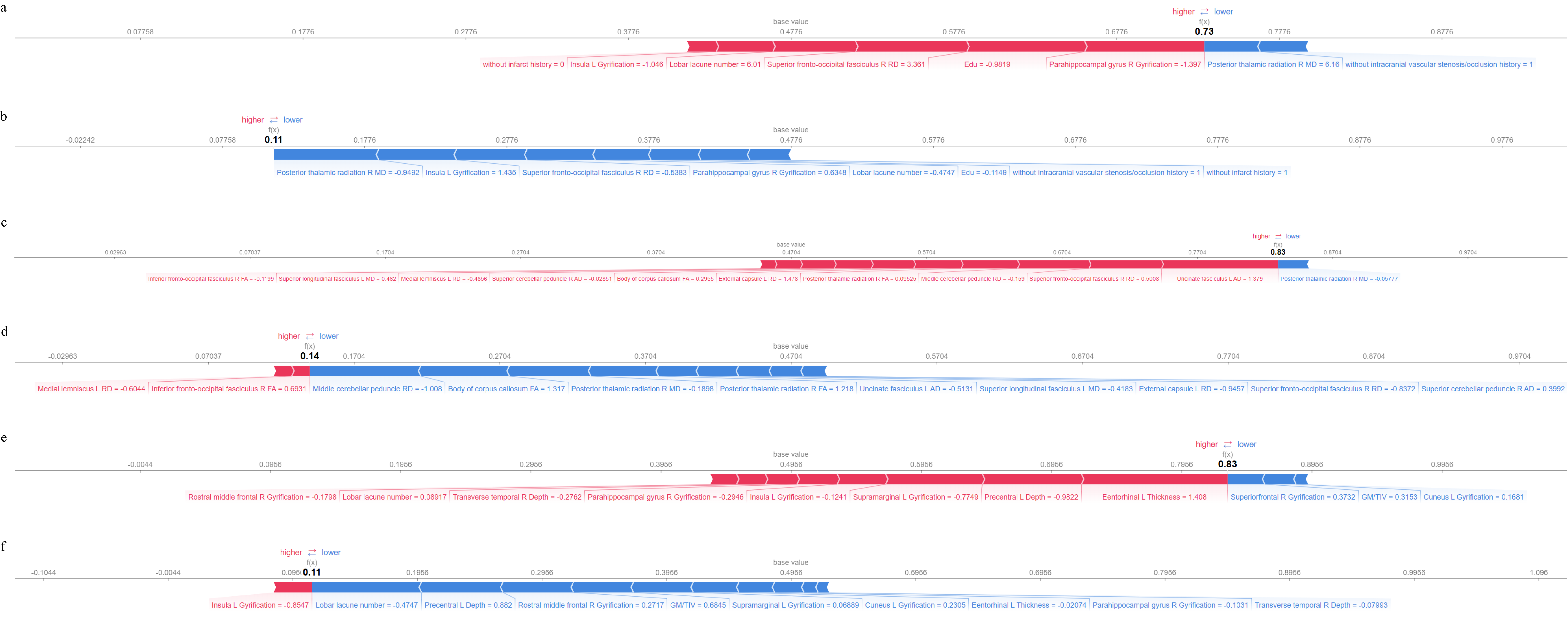


1. **Supplementary Tables**
   1. **Table S1: Demographics of external dataset**

Abbreviations: BMI, body mass index; CHD, coronary heart disease; SBP, systolic blood pressure; DBP, diastolic blood pressure; Fazekas-PV, Fazekas scale in periventricular whitter matter; EPVS, enlarged perivascular spaces; BG, basal ganglia; CSO, centrum semiovale.

| Demographics | Derivation cohort number (%) or mean (SD, score range) | |
| --- | --- | --- |
|  | Patients with VCI | Participants with normal cognition |
| Age(years) | 67.00 (4.36, 57-76) | 62.24 (5.19, 52-70) |
| Education (years) | 11.42 (2.49, 7-19) | 12.56 (2.14, 8-16) |
| Sex, female, n (%) | 36 (63.16%) | 10 (40.00%) |
| BMI (kg/m2) | 25.57 (4.03, 18.70-45.30） | 25.37 (3.04, 21.10-34.40） |
| MMSE | 26.28 (2.31, 20-30) | 27.96(1.49, 24-30) |
| MoCA | 20.61 (2.77, 13-25) | 24.76(1.96, 20-29) |
| Smoke, n (%) | 21 (36.84%) | 11 (44.00%) |
| Exercise, n (%) | 32 (56.14%) | 16 (64.00%) |
| Intracranial vascular stenosis/occlusion history, n (%) | 28 (49.12%) | 4 (16.00%) |
| Infarct history, n (%) | 10 (17.54%) | 0 (0%) |
| CHD, n (%) | 8 (14.04%) | 2 (8.00%) |
| Hypertension history, n (%) | 56 (98.25%) | 21 (84.00%) |
| Diabetes history, n (%) | 32 (56.14%) | 13 (52.00%) |
| Hyperlipidemia history, n (%) | 43 (75.44%) | 22 (88.00%) |
| SBP | 139.63 (14.15, 113-183) | 128.00 (16.33, 75-152) |
| DBP | 84.67 (8.96, 65-110) | 83.22 (10.89, 64-105.5) |
| CSO-EPVS | 0.05 (0.23, 0-1) | 0.04 (0.20, 0-1) |
| BG-EPVS | 0.77 (0.54, 0-2) | 0.72 (0.46, 0-1) |
| Deep lacune | 0.07 (0.26, 0-1) | 0.00 (0.00, 0) |
| Lobar lacune | 0.35 (1.89, 0-14) | 0.04 (0.20, 0-1) |
| Fazekas-PV | 1.93 (1.00, 0-3) | 0.87 (0.84, 0-3) |
| Fazekas-DEEP | 1.64 (1.02, 0-3) | 0.80 (0.75, 0-3) |

- 1. **Table S2: Top 10 performing models identified by Lazy Predict**

| Model | Balanced Accuracy | AUC | F1-Score |
| --- | --- | --- | --- |
| RandomForestClassifier | 0.91 | 0.91 | 0.90 |
| NearestCentroid | 0.90 | 0.90 | 0.90 |
| GaussianNB | 0.89 | 0.89 | 0.90 |
| BernoulliNB | 0.89 | 0.89 | 0.89 |
| SVC | 0.86 | 0.86 | 0.89 |
| ExtraTreesClassifier | 0.86 | 0.86 | 0.86 |
| NuSVC | 0.86 | 0.86 | 0.86 |
| XGBClassifier | 0.86 | 0.86 | 0.86 |
| QuadraticDiscriminantAnalysis | 0.86 | 0.86 | 0.86 |
| AdaBoostClassifier | 0.85 | 0.85 | 0.84 |

- 1. **Table S3 Performance comparison of initial 34-feature models constructed with different ML algorithms (All data modalities)**

Performance comparison of initial models, incorporating all 34 features combining clinical data and measures from T1, T2-FLAIR, and DTI, and constructed with various ML algorithms.

Abbreviations:95% CIs. AP: average precision. LR, logistic regression; RF, random forest; SVM, support vector machine; XGB, eXtreme gradient boosting; GNB, gaussian naive bayes.

| Models | AUC | Accuracy | Precision | Sensitivity | Specificity | F1-score | AP |
| --- | --- | --- | --- | --- | --- | --- | --- |
| XGB | 0.931 [0.864 - 0.982] | 0.873 [0.794 - 0.937] | 0.906 [0.824 - 0.973] | 0.853 [0.750 - 0.947] | 0.897 [0.800 - 0.971] | 0.879 [0.800 - 0.943] | 0.940 [0.875 - 0.986] |
| SVM | 0.907 [0.840 - 0.961] | 0.762 [0.667 - 0.841] | 0.880 [0.773 - 0.969] | 0.647 [0.500 - 0.784] | 0.897 [0.800 - 0.972] | 0.746 [0.632 - 0.839] | 0.915 [0.844 - 0.972] |
| LR | 0.913 [0.849 - 0.969] | 0.841 [0.762 - 0.921] | 0.929 [0.846 - 1.0] | 0.765 [0.643 - 0.886] | 0.931 [0.846 - 1.0] | 0.839 [0.750 - 0.917] | 0.922 [0.854 - 0.977] |
| RF | 0.962 [0.919 - 0.993] | 0.873 [0.794 - 0.937] | 0.933 [0.852 - 1.0] | 0.824 [0.710 - 0.927] | 0.931 [0.846 - 1.0] | 0.875 [0.793 - 0.943] | 0.970 [0.937 - 0.994] |
| GNB | 0.941 [0.888 – 0.984] | 0.889 [0.825 – 0.952] | 0.935 [0.857 – 1.0] | 0.853 [0.743 – 0.946] | 0.931 [0.846 – 1.0] | 0.892 [0.816 – 0.955] | 0.955 [0.912 – 0.990] |

- 1. **Table S4 Performance comparison of initial 18-feature models constructed with different ML algorithms (DTI)**

Performance comparison of initial models constructed with different machine learning algorithms using measures extracted from DTI.

| Models | AUC | Accuracy | Precision | Sensitivity | Specificity | F1-score | AP |
| --- | --- | --- | --- | --- | --- | --- | --- |
| XGB | 0.840 [0.748 - 0.917] | 0.778 [0.698 - 0.873] | 0.727 [0.622 - 0.833] | 0.941 [0.872 - 1.0] | 0.586 [0.429 - 0.739] | 0.821 [0.742 - 0.894] | 0.854 [0.750 - 0.934] |
| SVM | 0.873 [0.796 - 0.940] | 0.777 [0.683 - 0.857] | 0.917 [0.813 – 1.00] | 0.647 [0.514 - 0.774] | 0.931 [0.846 – 1.0] | 0.759 [0.643 - 0.852] | 0.902 [0.930 - 0.959] |
| LR | 0.887 [0.807 - 0.954] | 0.778 [0.683 - 0.857] | 0.885 [0.778 – 0.968] | 0.677 [0.543 - 0.806] | 0.897 [0.800 – 0.971] | 0.767 [0.655 - 0.853] | 0.905 [0.827 - 0.964] |
| RF | 0.897 [0.817 - 0.953] | 0.778 [0.683 - 0.857] | 0.885 [0.769 – 0.964] | 0.677 [0.533 - 0.806] | 0.897 [0.788 – 0.970] | 0.767 [0.654 - 0.857] | 0.920 [0.852 - 0.966] |
| GNB | 0.904 [0.830 - 0.966] | 0.714 [0.619 - 0.794] | 1.0 [1.0 - 1.0] | 0.471 [0.325 - 0.607] | 1.0 [1.0 - 1.0] | 0.640 [0.491 - 0.756] | 0.922 [0.856 - 0.972] |

- 1. **Table S5 Performance comparison of initial 14-feature models constructed with different ML algorithms (T1 & T2-FLAIR)**

Performance comparison of initial models constructed with different machine learning algorithms using measures extracted from T1 and T2-FLAIR.

| Models | AUC | Accuracy | Precision | Sensitivity | Specificity | F1-score | AP |
| --- | --- | --- | --- | --- | --- | --- | --- |
| XGB | 0.868 [0.788 - 0.943] | 0.810 [0.730 - 0.889] | 0.824 [0.714 - 0.923] | 0.824 [0.719 - 0.921] | 0.793 [0.667 - 0.912] | 0.824 [0.730 - 0.902] | 0.869 [0.779 - 0.956] |
| SVM | 0.853 [0.767 - 0.928] | 0.794 [0.714 - 0.873] | 0.839 [0.724 - 0.939] | 0.765 [0.633 - 0.879] | 0.828 [0.704 - 0.936] | 0.800 [0.696 - 0.880] | 0.886 [0.810 - 0.951] |
| LR | 0.864 [0.781 - 0.938] | 0.794 [0.714 - 0.873] | 0.862 [0.750 – 0.964] | 0.735 [0.600 - 0.853] | 0.862 [0.743 – 0.964] | 0.794 [0.689 - 0.877] | 0.887 [0.811 - 0.956] |
| RF | 0.866 [0.786 - 0.940] | 0.794 [0.714 - 0.873] | 0.818 [0.700 – 0.925] | 0.794 [0.677 - 0.903] | 0.793 [0.667 – 0.913] | 0.806 [0.708 - 0.885] | 0.866 [0.774 - 0.954] |
| GNB | 0.876 [0.794 - 0.950] | 0.825 [0.746 - 0.905] | 0.871 [0.767 - 0.967] | 0.794 [0.676 - 0.905] | 0.862 [0.750 - 0.963] | 0.831 [0.737 - 0.912] | 0.881 [0.793 - 0.963] |

- 1. **Table S6 Performance of the final models in external dataset**

△AUC and *p*: the difference of AUC and significance level between the external validation and internal validation of the final models.

| Modalities | Clinical data + T1 and T2-FLAIR + DTI | DTI | T1 and T2-FLAIR |
| --- | --- | --- | --- |
| △AUC and P | △AUC = 0.038 *p* = 0.308 | △AUC = 0.114 *p* = 0.108 | △AUC = 0.301 *p* < 0.001 |
| AUC | 0.919 [0.866 – 0.966] | 0.779 [0.674 – 0.865] | 0.571 [0.457 - 0.677] |
| AP | 0.966 [0.940 – 0.987] | 0.876 [0.798 – 0.943] | 0.783 [0.695 - 0.869] |
| F1-score | 0.857 [0.789 – 0.916] | 0.804 [0.722 – 0.868] | 0.609 [0.494 - 0.695] |
| Accuracy | 0.817 [0.744 – 0.890] | 0.744 [0.659 – 0.817] | 0.561 [0.463 - 0.646] |
| Precision | 0.938 [0.880 – 0.984] | 0.860 [0.776 – 0.939] | 0.800 [0.679 - 0.909] |
| Recall | 0.789 [0.691 – 0.875] | 0.754 [0.649 – 0.845] | 0.491 [0.373 - 0.59] |
| Specificity | 0.880 [0.769 – 0.972] | 0.720 [0.565 – 0.864] | 0.720 [0.556 - 0.864] |

- 1. **Table S7 Correlations between discriminative imaging measures and neuropsychological assessments within VCI group**

Correlations between discriminative imaging measures and neuropsychological assessments within VCI group, controlled by sex, age and education, corrected by FDR. GM, gray matter; TIV, total intracranial volume

| Region | MoCA | | MMSE | | TMT-A | | TMT-B | |
| --- | --- | --- | --- | --- | --- | --- | --- | --- |
|  | **R** | ***p_FDR_*** | **R** | ***p_FDR_*** | **R** | ***p_FDR_*** | **R** | ***p_FDR_*** |
| Lobar Lacune | -0.342 | **<0.001** | -0.243 | **0.009** | 0.252 | **0.012** | 0.273 | **0.006** |
| GM/TIV | 0.227 | **0.033** | 0.297 | **0.001** | -0.217 | **0.032** | -0.306 | **0.003** |
| Gyrification Parahippocampal R | -0.006 | 0.963 | -0.028 | 0.806 | -0.013 | 0.887 | 0.065 | 0.510 |
| Gyrification Cuneus L | 0.077 | 0.680 | 0.106 | 0.351 | -0.142 | 0.158 | -0.141 | 0.171 |
| Gyrification Insula L | 0.105 | 0.532 | 0.128 | 0.226 | -0.272 | **0.009** | -0.265 | **0.007** |
| Gyrification Supramarginal L | 0.086 | 0.630 | 0.175 | 0.074 | -0.150 | 0.141 | -0.137 | 0.176 |
| Gyrification Rostral middle frontal R | 0.087 | 0.630 | 0.325 | **<0.001** | -0.393 | **<0.001** | -0.351 | **<0.001** |
| Gyrification Superior-frontal R | 0.205 | **0.048** | 0.332 | **<0.001** | -0.305 | **0.003** | -0.281 | **0.006** |
| Thickness Frontal pole R | 0.017 | 0.963 | 0.188 | 0.062 | -0.194 | 0.060 | -0.166 | 0.109 |
| Depth Precentral L | -0.004 | 0.963 | 0.020 | 0.841 | -0.182 | 0.070 | -0.159 | 0.121 |
| Depth Transverse temporal R | -0.028 | 0.947 | -0.009 | 0.914 | -0.172 | 0.085 | -0.195 | 0.053 |
| FA Body of corpus callosum | 0.054 | 0.850 | 0.072 | 0.550 | -0.114 | 0.229 | -0.080 | 0.425 |
| FA Inferior fronto-occipital fasciculus R | -0.034 | 0.947 | -0.066 | 0.572 | -0.048 | 0.646 | -0.025 | 0.780 |
| FA Posterior thalamic radiation R | 0.129 | 0.350 | 0.131 | 0.226 | -0.249 | **0.012** | -0.201 | **0.048** |
| MD Posterior thalamic radiation R | -0.039 | 0.947 | -0.078 | 0.550 | 0.128 | 0.185 | 0.118 | 0.233 |
| MD Superior longitudinal fasciculus L | -0.315 | **<0.001** | -0.346 | **<0.001** | 0.258 | **0.012** | 0.316 | **0.003** |
| AD Superior cerebellar peduncle R | -0.011 | 0.963 | -0.056 | 0.578 | -0.020 | 0.860 | 0.057 | 0.547 |
| AD Uncinate fasciculus L | -0.197 | 0.053 | -0.263 | **0.004** | 0.319 | **0.002** | 0.225 | **0.027** |
| RD External capsule L | -0.219 | **0.035** | -0.180 | 0.071 | 0.253 | **0.012** | 0.273 | **0.006** |
| RD Medial lemniscus L | 0.006 | 0.963 | 0.055 | 0.578 | 0.131 | 0.185 | 0.110 | 0.260 |
| RD Middle cerebellar peduncle | -0.069 | 0.724 | -0.074 | 0.550 | 0.190 | 0.061 | 0.203 | **0.048** |
| RD Superior fronto-occipital fasciculus R | -0.032 | 0.947 | -0.058 | 0.578 | 0.127 | 0.185 | 0.127 |  |

1. **References**

1. Gaser C, Dahnke R, Thompson PM, Kurth F, Luders E, Initiative AsDN. Cat – a computational anatomy toolbox for the analysis of structural mri data. 2022:2022.2006.2011.495736

2. Friston KJ. *Statistical parametric mapping : The analysis of funtional brain images*. Amsterdam ; Boston: Elsevier/Academic Press; 2007.

3. Rolls ET, Huang CC, Lin CP, Feng J, Joliot M. Automated anatomical labelling atlas 3. *Neuroimage*. 2020;206:116189

4. Yotter RA, Dahnke R, Thompson PM, Gaser C. Topological correction of brain surface meshes using spherical harmonics. *Hum Brain Mapp*. 2011;32:1109-1124

5. Dahnke R, Yotter RA, Gaser C. Cortical thickness and central surface estimation. *Neuroimage*. 2013;65:336-348

6. Luders E, Thompson PM, Narr KL, Toga AW, Jancke L, Gaser C. A curvature-based approach to estimate local gyrification on the cortical surface. *Neuroimage*. 2006;29:1224-1230

7. Yotter RA, Nenadic I, Ziegler G, Thompson PM, Gaser C. Local cortical surface complexity maps from spherical harmonic reconstructions. *Neuroimage*. 2011;56:961-973

8. Desikan RS, Segonne F, Fischl B, Quinn BT, Dickerson BC, Blacker D, et al. An automated labeling system for subdividing the human cerebral cortex on mri scans into gyral based regions of interest. *Neuroimage*. 2006;31:968-980

9. Cui Z, Zhong S, Xu P, He Y, Gong G. Panda: A pipeline toolbox for analyzing brain diffusion images. *Front Hum Neurosci*. 2013;7:42

10. Hua K, Zhang J, Wakana S, Jiang H, Li X, Reich DS, et al. Tract probability maps in stereotaxic spaces: Analyses of white matter anatomy and tract-specific quantification. *Neuroimage*. 2008;39:336-347
